# The pitfalls of bedside regional cerebral oxygen saturation in the early stage of post cardiac arrest

**DOI:** 10.1186/s13049-015-0173-4

**Published:** 2015-11-11

**Authors:** Kosaku Kinoshita, Atsushi Sakurai, Shingo Ihara

**Affiliations:** Division of Emergency and Critical Care Medicine, Department of Acute Medicine, Nihon University School of Medicine, 30-1 Oyaguchi Kamimachi Itabashi-ku, Tokyo, 173-8610 Japan

**Keywords:** Cardiac arrest, Post-cardiac arrest patients, Regional cerebral oxygen saturation, Amplitude-integrated electroencephalography

## Abstract

It remains uncertain whether neuromonitoring reliably predicts outcome in adult post-cardiac arrest patients in the early stage treated with therapeutic hypothermia. Recent reports demonstrated a regional cerebral oxygen saturation of cardiac arrest patients on hospital arrival could predict their neurological outcome. There has been little discussion about the significance of regional cerebral oxygen saturation in patients with post-cardiac arrest syndrome. Amplitude-integrated electroencephalography monitoring may also provide early prognostic information for post-cardiac arrest syndrome. However, even when the initial electroencephalography is flat after the return of spontaneous circulation, good neurological outcome may still be obtainable if the electroencephalography shifts to a continuous pattern. The electroencephalography varied from flat to various patterns, such as flat, epileptic, or continuous during the first 24 h, while regional cerebral oxygen saturation levels varied even when the electroencephalography was flat. It is therefore difficult to estimate whether regional cerebral oxygen saturation accurately indicates the coupling of cerebral blood flow and metabolism in the early stage after cardiac arrest. Careful assessment of prognosis is necessary when relying solely on regional cerebral oxygen saturation as a single monitoring modality.

## Background

Recent reports demonstrate a relationship between regional cerebral oxygen saturation (rSO_2_) of cardiac arrest patients on hospital arrival and their neurological outcome [1, 2]. Data are insufficient to support the utility of neuromonitoring for the prediction of outcome of post-cardiac arrest syndrome (PCAS) patients [3] treated with therapeutic hypothermia. Few reports have discussed the sequential changes or physiological significance of rSO_2_ during therapeutic hypothermia immediately after the return of spontaneous circulation (ROSC). Monitoring with electroencephalography (EEG) may be able to provide early prognostic information after ROSC in patients with therapeutic hypothermia [4, 5]. Even when EEG indicates a flat pattern in the early stage of ROSC, good neurological outcome may still be obtainable after cardiac arrest if the EEG shifts to a continuous pattern during hypothermia [6]. Yet rSO_2_ values tend to vary widely even when EEG patterns are flat in the early stage, which makes it difficult to estimate the neurological outcome using only a single modality for monitoring by rSO_2_.

In this paper we will discuss the clinical pitfalls of rSO_2_ performed in conjunction with EEG for comatose patients after ROSC.

## Clinical issues of rSO_2_ in patients with PCAS

**Fig. 1:**
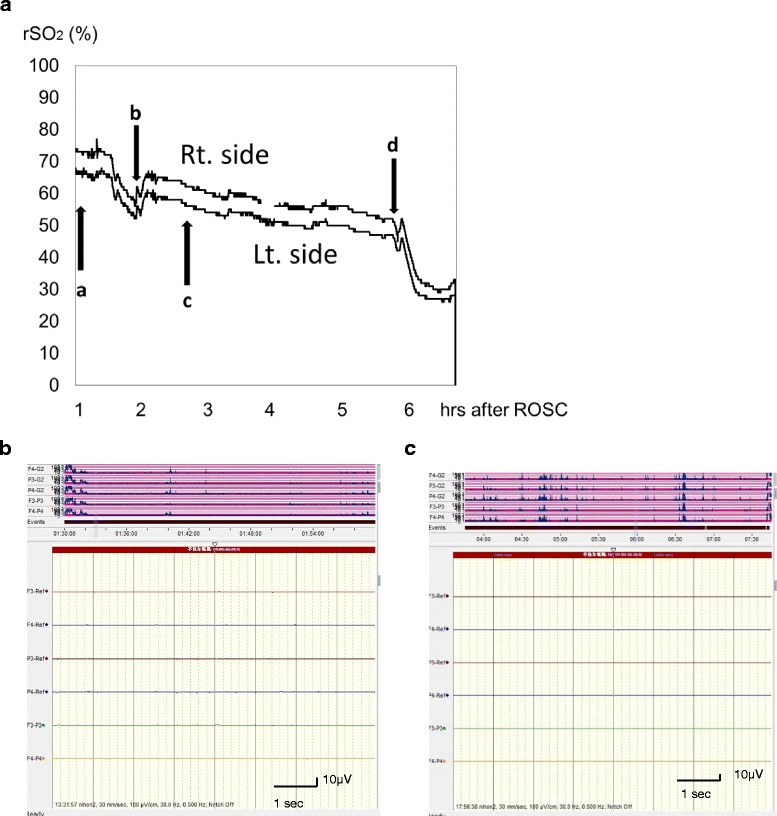
Changes in rSO_2_ and flat aEEG pattern in PCAS. A female in her 70s was transferred to the hospital by ambulance with an airway obstruction suffered during a meal. Her initial cardiac rhythm indicated pulseless electrical activity. The time from cardiac arrest to ROSC was 31 min. The initial rSO_2_value was 66–75 % (**a**-**a**) one hour after ROSC and the aEEG pattern was flat. Her rSO_2_ decreased with a decline in blood pressure and rose following dopamine infusion (**a**-**b**). However, her rSO_2_ gradually decreased (**a**-**c**) and she experienced another cardiac arrest (*a*-*d*) with a steep decline in rSO_2_. Throughout the course of treatment in the ICU, the aEEG maintained a flat pattern regardless of rSO_2_ value (**b** and **c**). A: rSO_2_ (%); B: aEEG, 1 h after ROSC. C: aEEG, 5 h after ROSC. Arrows: (**a**): BP 112/58 mmHg, arterial gases: PaCO_2_ 56.8 mmHg, pH 7.189; (**b**): sBP 88 mmHg, start dopamine administration, PaCO_2_ 42.6 mmHg, pH 7.232; (**c**): sBP 92 mmHg, arterial gases: PaCO_2_ 39.6 mmHg, pH 7.353; *d*: cardiac arrest, start chest compression. sBP: systolic blood pressure; ROSC: return of spontaneous circulation; rSO_2_: regional cerebral oxygen saturation; aEEG: amplitude-integrated electroencephalography (NicoletOne^TM^ IMI, Japan)

Theoretically, rSO_2_ can estimate the balance between the cerebral metabolic rate of oxygen (CMRO_2_) and cerebral blood flow (CBF), which linearly correlates with cerebral venous oxygen saturation and with CBF [7]. A host of factors such as blood pressure, blood volume, blood viscosity, oxygen delivery/metabolism, and hypo/hypercapnia lead to the vasodilation or constriction of the brain vessels when the cerebral autoregulatory mechanism responds normally, [8] and cerebral autoregulation keeps the CBF constant in response to changes in these factors. However, the conditions of PCAS have many factors for dysautoregulation such as hypotension and increase or decrease in PaCO_2_, whereby CBF would be affected by these dysautoregulatory factors. Physicians therefore have to consider many factors when estimating rSO_2_ values after cardiac arrest. The rSO_2_ data of brain-dead patients also demonstrates that rSO_2_ values do not always indicate the cerebral oxygen metabolism [9]. As seen in Fig. 1a, for example, an aEEG pattern can remain flat regardless of the rSO_2_ value. The rSO_2_ value may depend on the blood pressure, because CMRO_2_ is thought be extremely suppressed when the EEG pattern is flat (Fig. 1b, c).

In the experimental model, the EEG was flat after a transient occlusion of both common carotid arteries and gradually changed from a flat to a continuous pattern after the release of the occlusion [10]. In the clinical setting, the EEG is also flat after cardiac arrest and changed from a flat to a various patterns after ROSC [6]. CMRO_2_ was also initially depressed after ROSC [11, 12]. The balance of CBF and metabolism is significantly altered after ROSC [13] while CBF might be normal [14] or decreased [11] in the resuscitated brain during the 24 h following cardiac arrest. A previous study reports that CMRO_2_ is not able to predict neurological outcome in the early stage of ROSC [15]. Therefore, the estimation of injured brain or outcome using for rSO_2_ might need to be carefully assessed in the early stage after PCAS.

## Higher rSO_2_ in the early stage in PCAS

**Fig. 2:**
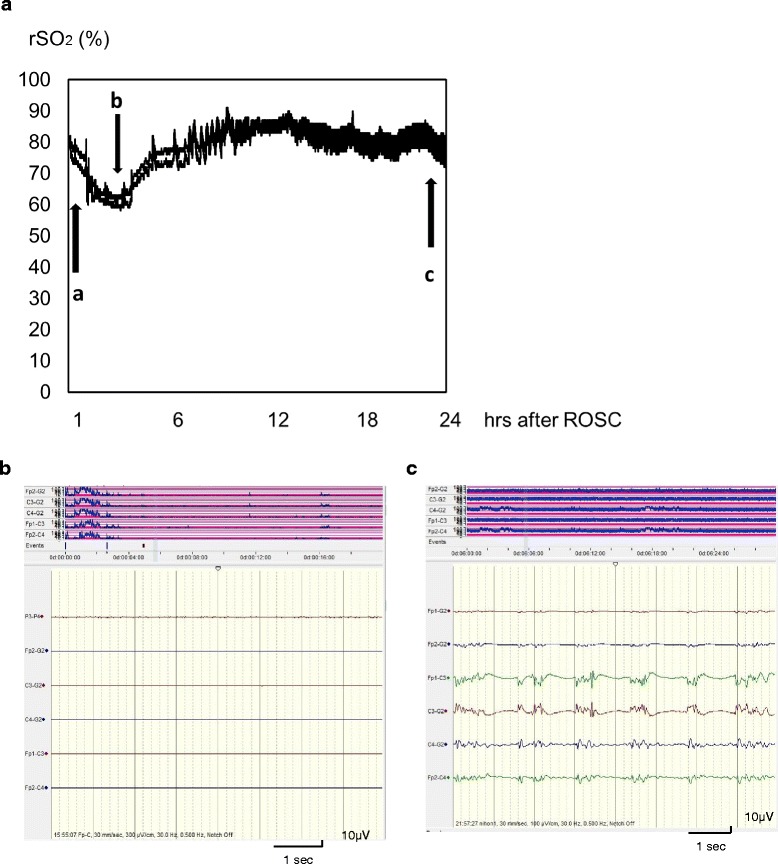
High rSO_2_ and epileptic aEEG pattern in PCAS. A male in his 50s collapsed suddenly with chest pain. His initial cardiac rhythm indicated ventricular fibrillation. The time from cardiac arrest to ROSC was 24 min. The diagnosis for this patient was acute myocardial infarction. The rSO_2_ monitoring and concurrent aEEG monitoring was commenced an hour after his collapse. His initial rSO_2_ reading was 80 % (**a**-**a**) and the aEEG pattern (**b**) was flat regardless of rSO_2_ value. Therapeutic hypothermia was commenced after the patient’s ICU admission and his rSO_2_ gradually decreased as his systemic arterial pressure fell. The patient’s rSO_2_ returned to its initial level once he received a dopamine infusion (**a**-**b**). The aEEG pattern changed to epileptic approximately 24 h after ROSC (**c**). The outcome for this patient was a persistent vegetative state. **a** rSO_2_ (%); B: aEEG, 1 h after ROSC; C: aEEG, 25 h after ROSC. Arrows: a: BP 168/100 mmHg, arterial gases: PaCO_2_ 34.7 mmHg, pH 7.441; b: BP 85/40 mmHg, start of dopamine administration, arterial gases: PaCO_2_ 34.2 mmHg, pH 7.429; c: BP 136/88 mmHg, arterial gases: PaCO_2_ 38.2 mmHg, pH 7.399. BP: blood pressure; ROSC: return of spontaneous circulation; rSO_2_: regional cerebral oxygen saturation; aEEG: amplitude-integrated electroencephalography

**Fig. 3:**
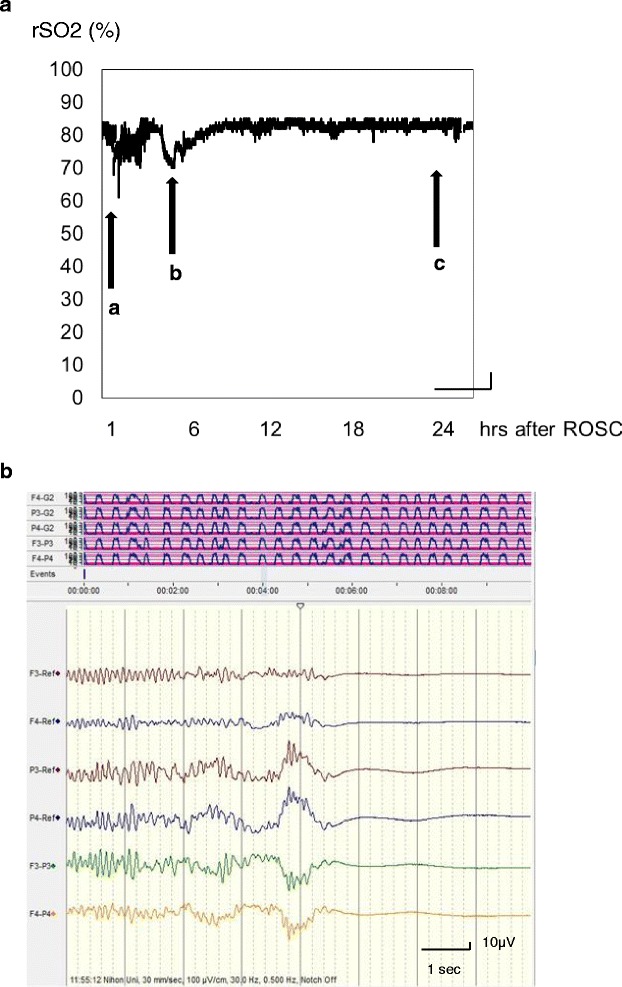
High rSO_2_ and suppression-burst aEEG pattern in PCAS. A female in her 30s experienced cardiac arrest caused by severe bronchial asthma. Her initial cardiac rhythm indicated pulseless electrical activity. The time from cardiac arrest to ROSC was 45 min. The initial rSO_2_value was 85 % (Fig. 4a-a). The aEEG pattern indicated a suppression-burst pattern 3 h after ROSC. She received a dopamine infusion after a fall in blood pressure (Fig. 4a-b). Thereafter, her rSO_2_ gradually returned around 80–85 % and kept constant (Fig. 1a-c) for 24 h. The outcome for this patient was brain death. aEEG measurements were taken for only 3 h after ROSC (not for 24 h) in this patient. (**a**): rSO_2_ (%); (**b**): aEEG, 3 h after ROSC. Arrows: a: BP 155/94 mmHg, arterial gases: PaCO_2_ 77.2 mmHg, pH 7.125; b: BP 90/48 mmHg, arterial gases: PaCO_2_ 44.1 mmHg, pH 7.430; c: BP 124/54 mmHg, arterial gases: PaCO_2_ 39.4 mmHg, pH 7.455. BP: blood pressure; ROSC: return of spontaneous circulation; rSO_2_: regional cerebral oxygen saturation; aEEG: amplitude-integrated electroencephalography

Higher rSO_2_ values can generally be assumed to have the following pathophysiological significance: hyperemia, including reactive hyperemia (e.g., an increased level of PaCO_2_), or hyperperfusion (lower CMRO_2_ and higher CBF). They can also reflect hyperemia caused by severe metabolic depression due to severe brain damage in PCAS. Cerebral oxygen extraction fraction, however, can be expected to decrease in comatose patients immediately after ROSC as a consequence of the primary cerebral metabolic suppression [11]. This appears to be the case even if higher rSO_2_ levels are detected when the EEG is flat, given that the CMRO_2_ may be drastically suppressed. Figures 2 and 3 demonstrate an rSO_2_ level that starts at around 80 % and remains constantly high thereafter. Finally, the aEEG indicated epileptic or suppression-burst EEG patterns of a type thought to portend poor outcome. It was difficult to determine the clinical significance of the high rSO_2_ values in the early post-resuscitation phase [4–6]. We therefore suspect that high rSO_2_ values with epileptic or suppression-burst EEG patterns indicate more severe brain damage after cardiac arrest.

## Lower rSO_2_ in the early stage in PCAS

Meanwhile, other factors cause lower rSO_2_ values in PCAS. The main causes of low rSO_2_ stem from too little oxygen supply to meet the cerebral oxygen demand, a sign of cerebral ischemia caused by unstable hemodynamics, hypoxia, or decreased PaCO_2_ rather than a cerebral metabolic suppression. Physicians will be able glean hints for the next steps in their treatment strategies for conditions of these types. Good neurological outcome may be obtainable after PCAS if two conditions are met: first, the patient shows no drastic elevation of rSO_2_ accompanying an extremely low voltage on the initial EEG; second, the patient shows and no sign of electrographic status epilepticus (Fig. 2) or a suppression-burst pattern (Fig. 3), especially in the early post-resuscitation phase [6].

A second cause of lower rSO_2_ values is the continuation of a no-reflow phenomenon suffered by the brain, which is characterized by lack of reperfusion after cerebral ischemia [16] caused by post-ischemic hypoperfusion, an increase in blood viscosity, a reduction in the caliber of small vessels, or impaired microvascular perfusion [16, 17]. In the results, CBF might be reduced even if blood pressure is within a normal range. Interestingly, no-reflow areas manifested as spotty areas of persistently disturbed recirculation were found to spread from the deep site to the brain surface cortex as the duration of the cardiac arrest increased [16]. This was a point of concern, because the rSO_2_ probes are placed over the patient’s forehead. We suspect that the presence of a spotty no-reflow area on the brain surface may impede the assessment of the cerebral oxygen metabolism for rSO_2_ because it measures the hemoglobin oxygen saturation in the regional forebrain cortex.

## Continuous monitoring for rSO_2_ performed in conjunction with EEG for a PCAS patient undergoing therapeutic hypothermia

**Fig. 4:**
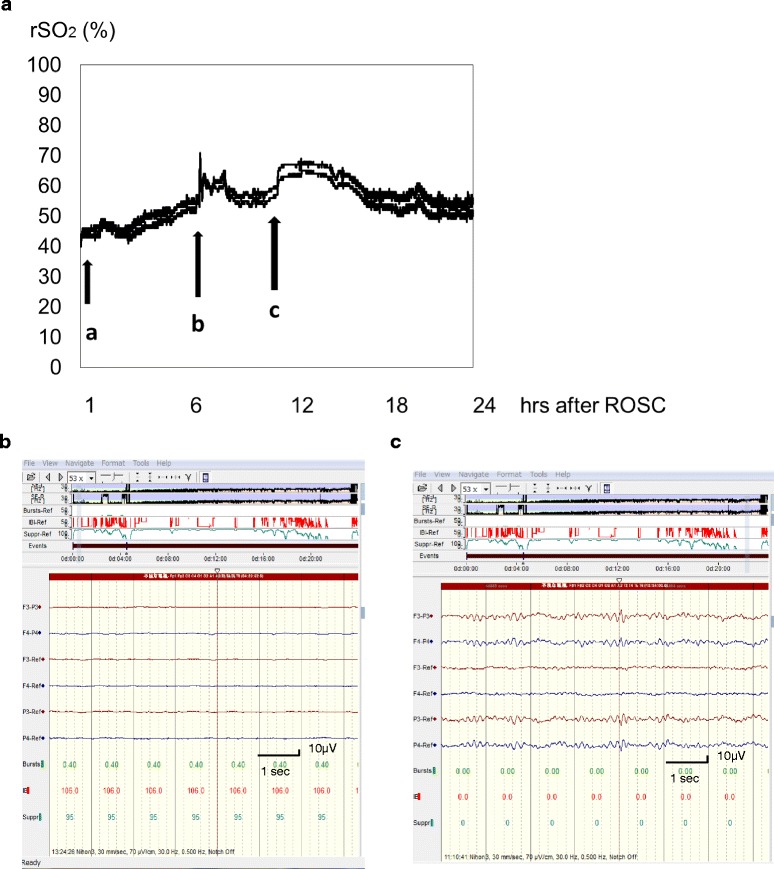
Changes in rSO_2_ and continuous aEEG pattern in PCAS. A male in his 50s collapsed suddenly while running. He received by-stander CPR and his initial cardiac rhythm indicated ventricular fibrillation. Defibrillation was performed by paramedics (34 min after collapse) and the patient was transferred to our hospital. ROSC was obtained and therapeutic hypothermia initiated immediately following his admission. The initial rSO_2_ value was around 45 % after ROSC. The aEEG pattern was flat at 3 h after ROSC (**b**) and shifted to a continuous pattern over the next 21 h (**c**). His final neurological outcome was favorable. **a** rSO_2_ (%); B: aEEG, 3 h after ROSC; (**c**): aEEG, 24 h after ROSC. Arrows; (**a**): BP 100/54 mmHg, arterial gases: PaCO_2_ 32.7 mmHg, pH 7.338; (**b**): BP 182/112 mmHg, arterial gases: PaCO_2_ 28.4 mmHg, pH 7.437; (**c**): BP 174/120 mmHg arterial gases: PaCO_2_ 36.6 mmHg, pH 7.400. BP: blood pressure; ROSC: return of spontaneous circulation; rSO_2_: regional cerebral oxygen saturation; aEEG: amplitude-integrated electroencephalography

There has been little discussion about the significance of rSO_2_ in patients with PCAS who are undergoing therapeutic hypothermia. The normal range of rSO_2_ in adult PCAS patients treated with therapeutic hypothermia also remains uncertain because of the hypothermic reduction of CMRO_2_. The amplitude-integrated electroencephalography (aEEG) in PCAS during therapeutic hypothermia was recently classified into four categories: extremely low voltage (flat; maximum voltage < 5 μV); suppression-burst pattern; electrographic status epilepticus with recurrent epileptic form activity; and continuous EEG [6]. Status epilepticus (Fig. 2) and suppression-burst patterns (Fig. 3) on EEG are suggested to have poor outcomes [4–6]. Patterns of these types could be used to predict extensive brain injury after cardiac arrest [4, 5]. Although the initial continuous aEEG pattern was flat (extremely low voltage), we were interested to observe cases in which the aEEG subsequently shifted to a continuous pattern over the following 24 h of therapeutic hypothermia (Fig. 4) and later went on to obtain a good neurological outcome [6]. During the time EEG is flat in the early stage after ROSC, the rSO_2_ values might be reflected by the influences from factors such as blood pressure, CBF, PaCO_2_ or no-reflow phenomenon. These result demonstrate that rSO_2_ alone is insufficient for predicting neurological outcome after PCAS and that rSO_2_ cannot be confirmed to reliably indicate whether CBF and metabolism are coupling or uncoupling in the early stage after ROSC.

## Conclusion

The EEG is extremely suppressed after ROSC and gradually changes to various patterns. CMRO_2_ might also be initially depressed while the EEG is suppressed. Given the possible therapeutic implications of continuous rSO_2_ monitoring in patients with brain injury, the variations of rSO_2_ and potential influences of many factors at this stage made it difficult to determine the clinical significance of rSO_2_ in PCAS. Careful assessment of prognosis is necessary when relying solely on rSO_2_ as a single monitoring modality.

## Patients consent

Informed consent was obtained from all individual participants included in the study.

This study was approved by the Clinical Research Institutional Review Board (IRB: RK-140613-3) of this hospital.

## Abbreviations

PCAS: Post-cardiac arrest syndrome
rSO_2_: Regional cerebral oxygen saturation
ROSC: Return of spontaneous circulation
EEG: Electroencephalography
CMRO_2_: Cerebral metabolic rate of oxygen
CBF: Cerebral blood flow
aEEG: Amplitude-integrated electroencephalography
